# Neoagaro-oligosaccharide monomers inhibit inflammation in LPS-stimulated macrophages through suppression of MAPK and NF-κB pathways

**DOI:** 10.1038/srep44252

**Published:** 2017-03-07

**Authors:** Wei Wang, Pei Liu, Cui Hao, Lijuan Wu, Wenjin Wan, Xiangzhao Mao

**Affiliations:** 1 College of Food Science and Engineering, and School of Medicine and Pharmacy, Ocean University of China, Qingdao, 266003, China; 2 Institute of Cerebrovascular Diseases, Affiliated Hospital of Qingdao University Medical College, Qingdao, 266003, China; 3 Department of Biology, Hong Kong Baptist University, Hong Kong, China

## Abstract

Neoagaro-oligosaccharides derived from agarose have been demonstrated to possess a variety of biological activities, such as anti-bacteria and anti-oxidative activities. In this study, we mainly explored the inhibitory effects and the mechanisms of neoagaro-oligosaccharide monomers against LPS-induced inflammatory responses in mouse macrophage RAW264.7 cells. The results indicated that neoagaro-oligosaccharide monomers especially neoagarotetraose could significantly reduce the production and release of NO in LPS-induced macrophages. Neoagarotetraose significantly suppressed the expression and secretion of inducible nitric oxide synthase (iNOS) and proinflammatory cytokines such as TNF-α and IL-6. The inhibition mechanisms may be associated with the inhibition of the activation of p38MAPK, Ras/MEK/ERK and NF-κB signaling pathways. Thus, neoagarotetraose may attenuate the inflammatory responses through downregulating the MAPK and NF-κB signaling pathways in LPS-stimulated macrophages. In summary, the marine-derived neoagaro-oligosaccharide monomers merit further investigation as novel anti-inflammation agents in the future.

Acute inflammation, which involves recruitment and activation of neutrophils, is a rapid response to infectious microbes or injured tissues^1^. The first line of defense of the immune system against infectious microbes are macrophages^2^, which can be activated through toll-like receptors (TLRs) signal pathway^3^,^4^. TLR4 ligation with the signal activator lipopolysaccharide (LPS) can induce activation of specific intracellular pathways including two pathways of mitogen-activated protein kinases (MAPKs) and nuclear factor-kB (NF-κB)^3^,^5^. The MAPKs pathway and NF-κB pathway then induce the expression of the inflammatory mediators such as inducible nitric oxide synthase (iNOS), and proinflammatory cytokines (tumor necrosis factor-α (TNF-α), interleukin-1β (IL-1β), and interleukin-6 (IL-6))^6^,^7^,^8^. Thus, compounds that can reduce the LPS-inducible inflammatory mediators may be developed into therapeutic agents for inflammation related diseases^8^.

Agarose, derived from red algae, is a neutral linear polysaccharide composed of alternating residues of 4-O-linked 3,6-anhydro-α-L-galactopyranose and 3-O-linked β-D-galactopyranose, and widely used in food, medicine, and biological research^9^,^10^. Agarose can be enzymatic hydrolyzed to generate agaro-oligosaccharides by α-agarase at the α-1,3 linkage and to generate neoagaro-oligosaccharides by β-agarase at the β-1,4 linkage^11^. In recent years, agaro-oligosaccharides and neoagaro-oligosaccharides have been reported to possess lots of biological activities, such as antioxidative activities^12^, moisturizing effect on skin^13^, and anti-inflammation effects^14^, which suggested that these oligosaccharides merit further investigation as functional foods to control inflammation.

In the current study, the inhibitory effects and mechanisms of neoagaro-oligosaccharides against LPS-induced inflammatory responses were investigated in mouse macrophage RAW 264.7 cells. The results showed that neoagaro-oligosaccharides especially neoagarotetraose effectively inhibited the inflammatory responses in RAW 264.7 cells mainly through downregulating the MAPK and NF-κB signaling pathways.

## Results

### Effects of neoagaro-oligosaccharide monomers on cell viability

**Figure 1:**
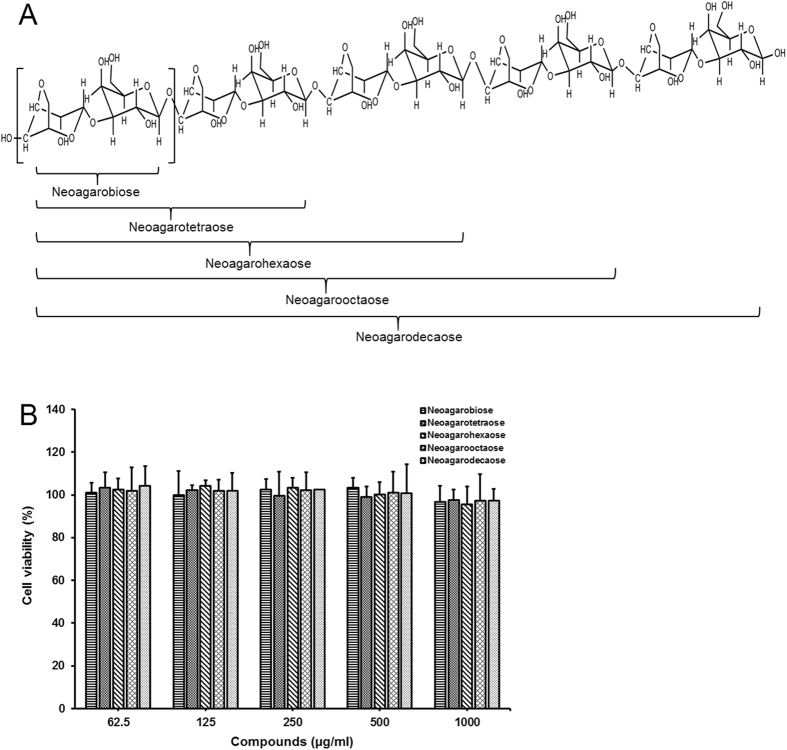
Chemical structure and cytotoxicity assay of neoagaro-oligosaccharide monomers. (**A**) Schematic illustration of neoagaro-oligosaccharides structure. The degree of polymerization (DP) of neoagaro-oligosaccharides is 2, 4, 6, 8, 10. (**B**) Cytotoxicity of neoagaro-oligosaccharides in RAW264.7 cells. RAW264.7 cells were cultured in the presence of neoagaro-oligosaccharides at indicated concentrations (62.5, 125, 250, 500, 1000 μg/ml) for 24 h. Then the cell viability was measured by MTT assay. Values are means ± S.D. (n = 3).

In this study, the neoagaro-oligosaccharide monomers neoagarobiose (NA2), neoagarotetraose (NA4), neoagarohexaose (NA6), neoagarooctaose (NA8), and neoagarodecaose (NA10) (≥98% purity) (Fig. 1A) were prepared in our laboratory as described previously^15^,^16^,^17^,^18^. The cytotoxicity of neoagaro-oligosaccharides (neoagarobiose, neoagarotetraose, neoagarohexaose, neoagarooctaose, neoagarodecaose) in RAW264.7 cells was evaluated by MTT assay. As shown in Fig. 1B, after exposure to neoagaro-oligosaccharides with different concentrations (62.5, 125, 250, 500 and 1000 μg/ml) for 24 h, the viabilities of RAW264.7 cells were all more than 95% of the PBS treated control group, which suggested that neoagaro-oligosaccharides at concentrations ranging from 62.5 to 1000 μg/ml had no significant cytotoxicity on RAW 264.7 cells. Therefore, concentrations of neoagaro-oligosaccharides were selected from 62.5 to 500 μg/ml for study on anti-inflammatory effects *in vitro*.

### Neoagarotetraose significantly decreased LPS-induced production of NO in RAW264.7 cells

**Figure 2:**
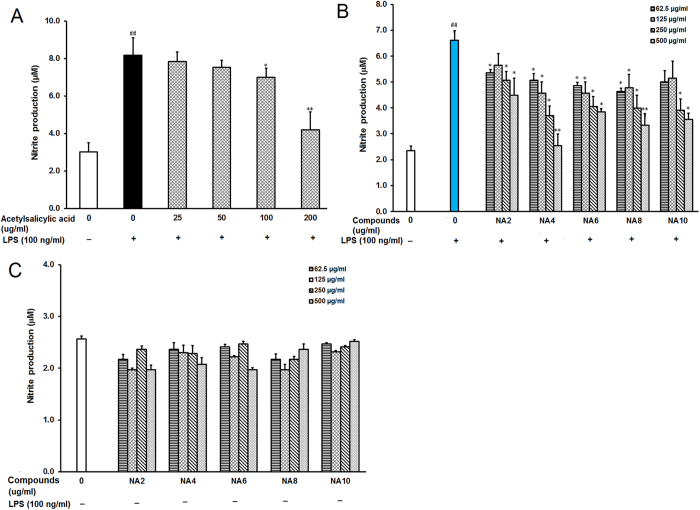
Effects of neoagaro-oligosaccharides on LPS-induced production of NO in RAW264.7 cells. (**A**) After pre-treatment of cells with different concentrations of acetylsalicylic acid (25, 50, 100, 200 μg/ml) for 2 h, the LPS (100 ng) was added to cells and incubated for 16 h. Then the content of nitrite in cell supernatant was determined by ELISA. Values are means ± SD (n = 3). Significance: ^##^P < 0.01 vs. normal control; *P < 0.05, **P < 0.01 vs. LPS treated control. (**B**) After treatment of cells with different neoagaro-oligosaccharide monomers (NA2, NA4, NA6, NA8, NA10) at indicated concentrations (62.5, 125, 250, 500 μg/ml) for 2 h and 100 ng LPS for 16 h, the content of nitrite in cell supernatant was measured by ELISA. Values are the mean ± SD (n = 3). Significance: ^#^P < 0.05, ^##^P < 0.01 vs. normal control; *P < 0.05, **P < 0.01 vs. LPS treated control. (**C**) After the treatment of cells with different neoagaro-oligosaccharide monomers (NA2, NA4, NA6, NA8, NA10) at indicated concentrations (62.5, 125, 250, 500 μg/ml) for 16 h, the content of nitrite in cell supernatant was measured by ELISA. Values are the mean ± SD (n = 3).

To evaluate the effects of neoagaro-oligosaccharides on the macrophage inflammatory responses induced by LPS, the NO production was evaluated by measuring the content of nitrite accumulated in culture medium based on the Griess reaction as previously described^8^. LPS treatment induced high nitrite production in RAW264.7 cells (Fig. 2A), while the positive control drug acetylsalicylic acid significantly decreased the nitrite production at the concentrations of 100 and 200 μg/ml (P < 0.05). Pretreatment with neoagaro-oligosaccharides (NA2, NA4, NA6, NA8, NA10) at indicated concentrations (62.5, 125, 250, 500 μg/ml) for 2 h markedly decreased the nitrite production in cell culture media compared to that in LPS treated control group (Fig. 2B). Neoagarotetraose (NA4), neoagarohexaose (NA6) and neoagarooctaose (NA8) also significantly decreased the nitrite production at low concentrations (62.5 and 125 μg/ml), and neoagarotetraose markedly decreased the nitrite production from 6.6 to about 2.5 μM at 500 μg/ml, superior to other neoagaro-oligosaccharide monomers (Fig. 2B). Thus, neoagarotetraose was used to further explore the inhibition mechanisms of neoagaro-oligosaccharides against LPS-induced inflammation responses in RAW264.7 cells.

Moreover, we also evaluated the effects of neoagaro-oligosaccharide monomers on RAW264.7 cells without LPS treatment, and found that neoagaro-oligosaccharide monomers did not significantly enhance the production of nitrite in RAW264.7 cells (Fig. 2C), suggesting that neoagaro-oligosaccharide monomers possess anti-inflammatory activities rather than the immune-stimulating activities in RAW264.7 cells.

### Neoagarotetraose decreased protein and mRNA levels of pro-inflammatory cytokines in LPS-stimulated RAW264.7 cells

**Figure 3:**
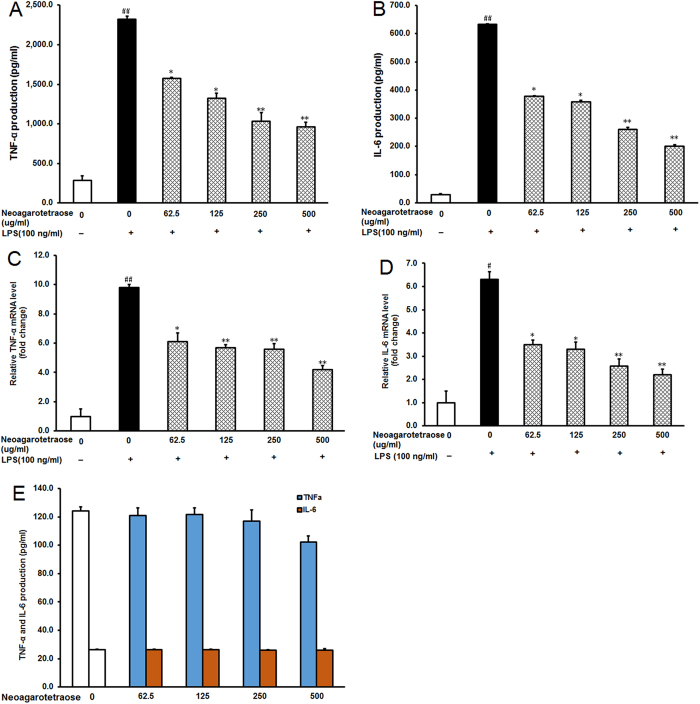
Neoagarotetraose decreased protein and mRNA levels of pro-inflammatory cytokines in LPS-stimulated RAW264.7 cells. (**A,B**) After the pre-treatment of cells with different concentrations of neoagarotetraose (62.5, 125, 250, 500 μg/ml) for 2 h, the LPS (100 ng) was added to cells and incubated for 16 h. Then the protein levels of pro-inflammatory cytokines TNF-α (**A**) and IL-6 (**B**) in cell culture media were measured using ELISA kits in a microplate reader, respectively. Values are the means ± SD (n = 3). Significance: ^##^P < 0.01 vs. normal control; *P < 0.05, **P < 0.01 vs. LPS treated control. (**C,D**) The total mRNAs of RAW264.7 cells were collected after treatment with neoagarotetraose (62.5, 125, 250, 500 μg/ml) for 2 h and 100 ng LPS for 16 h. Then the mRNA levels of TNF-α gene (**C**) and IL-6 gene (**D**) were detected by quantitative RT-PCR assay, respectively. The relative amounts of TNF-α and IL-6 mRNAs were determined using the comparative (2^−ΔΔCT^) method. The mRNA levels for non-drug treated cells (Control) were assigned values of 1. Values are means ± S.D. (n = 3). Significance: ^#^P < 0.05, ^##^P < 0.01 vs. normal control; *P < 0.05, **P < 0.01 vs. LPS treated control.

To further examine the inhibitory effects of neoagarotetraose on LPS-induced production of pro-inflammatory cytokines, the protein and mRNA levels of TNF-α and IL-6 in LPS treated RAW 264.7 cells were investigated by ELISA and quantitative RT-RCR, respectively. In normal control cells, TNF-α, and IL-6 were only slightly produced, but in response to LPS, TNF-α and IL-6 were significantly increased from 250 and 25 ng/L to about 2300 and 625 ng/L, respectively (P < 0.01) (Fig. 3A,B). However, pretreatment with neoagarotetraose (62.5, 125, 250, 500 μg/ml) significantly inhibited the LPS-induced TNF-α and IL-6 production in a concentration-dependent manner (Fig. 3A,B). Especially for the treatment of neoagarotetraose at 250 and 500 μg/ml, the production of TNF-α and IL-6 could be significantly reduced to less than 50% of that in LPS treated control group (P < 0.01) (Fig. 3A,B). Moreover, the inhibitory effects of neoagarotetraose on mRNA expression seemed to follow a similar pattern to those on protein production (Fig. 3C,D). The mRNA levels of TNF-α and IL-6 were significantly increased by LPS, while pretreatment with neoagarotetraose (62.5, 125, 250, 500 μg/ml) markedly and concentration-dependently reduced the mRNA levels of TNF-α and IL-6 from 10.0 and 6.0-fold to about 6.0 and 3.0-fold of normal control group, respectively (P < 0.05) (Fig. 3C,D). Thus, neoagarotetraose can reduce both the protein and mRNA levels of pro-inflammatory cytokines in LPS-stimulated macrophages.

Furthermore, we also tested the effects of neoagarotetraose on the levels of TNF-α and IL-6 in RAW264.7 cells without LPS treatment by ELISA, and the results showed that neoagarotetraose (62.5–500 μg/ml) treatment could not significantly inhibit the secretion of TNF-α and IL-6, as compared to the normal control group (Fig. 3E). Therefore, neoagarotetraose could specifically inhibit the expression and secretion of pro-inflammatory cytokines induced by LPS treatment.

### Neoagarotetraose decreased LPS-induced production of iNOS and IL-1β in RAW264.7 cells

**Figure 4:**
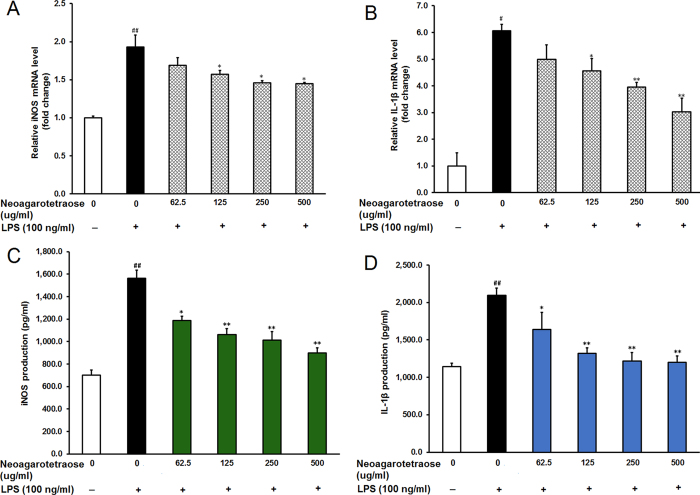
Neoagarotetraose decreased LPS-induced production of iNOS and IL-1β in RAW264.7 cells. (**A,B**) After pretreated with neoagarotetraose (62.5, 125, 250, 500 μg/ml) for 2 h, RAW264.7 cells were exposed to 100 ng LPS for 24 h. Then the mRNA levels of iNOS gene (**A**) and IL-1β gene (**B**) were detected by quantitative RT-PCR assay, respectively. The relative amounts of iNOS and IL-1β mRNAs were determined using the comparative (2^−ΔΔCT^) method. The mRNA levels for non-drug treated cells (Control) were assigned values of 1. Values are means ± S.D. (n = 3). Significance: ^#^P < 0.05, ^##^P < 0.01 vs. normal control; *P < 0.05, **P < 0.01 vs. LPS treated control. (**C,D**) After treatment, the protein levels of iNOS (**C**) and IL-1β (**D**) in cell culture media were measured using ELISA kits. Values are means ± SD (n = 3). Significance: ^##^P < 0.01 vs. normal control; *P < 0.05, **P < 0.01 vs. LPS treated control.

The high levels of NO induced by stimulation with LPS are often produced by the inducible isoform of the enzyme nitric oxide synthase (iNOS)^19^. Thus, we further explored whether the inhibitory effect of neoagarotetraose on NO production was due to inhibition of iNOS expression by using real time RT-PCR and ELISA assay. As shown in Fig. 4A, LPS treatment induced upregulation of iNOS mRNA expression while pretreatment with neoagarotetraose (62.5, 125, 250, 500 μg/ml) reduced iNOS mRNA expression in a dose-dependent manner. Neoagarotetraose significantly decreased the iNOS mRNA level when used at the concentration of >125 μg/ml (P < 0.05) (Fig. 4A). In addition, neoagarotetraose also significantly inhibited the LPS-induced iNOS protein expression in a dose-dependent manner at the concentrations of 125–500 μg/ml (P < 0.05), compared to that in LPS treated control cells (Fig. 4C).

Moreover, the inhibitory effects of neoagarotetraose on the mRNA and protein levels of another cytokine IL-1β were also evaluated by quantitative RT-PCR and ELISA assay. The results showed that the mRNA levels of IL-1β significantly increased upon LPS treatment but this induction was effectively inhibited by neoagarotetraose treatment in a dose-dependent manner (Fig. 4B). Neoagarotetraose could significantly reduce the mRNA expression of IL-1β when used at the concentration >125 μg/ml (P < 0.05) (Fig. 4B). Similar was the case of protein expression of IL-1β in LPS-induced RAW264.7 cells. Neoagarotetraose significantly suppressed the LPS-induced IL-1β protein expression in a dose-dependent manner at the concentrations of 125–500 μg/ml (P < 0.05), compared to that in LPS treated control cells (Fig. 4D). Thus, neoagarotetraose may reduce the production of NO through inhibiting the expression of pro-inflammatory mediators such as iNOS and IL-1β.

### Neoagarotetraose affected phosphorylation of MAPK in LPS-stimulated RAW 264.7 cells

**Figure 5:**
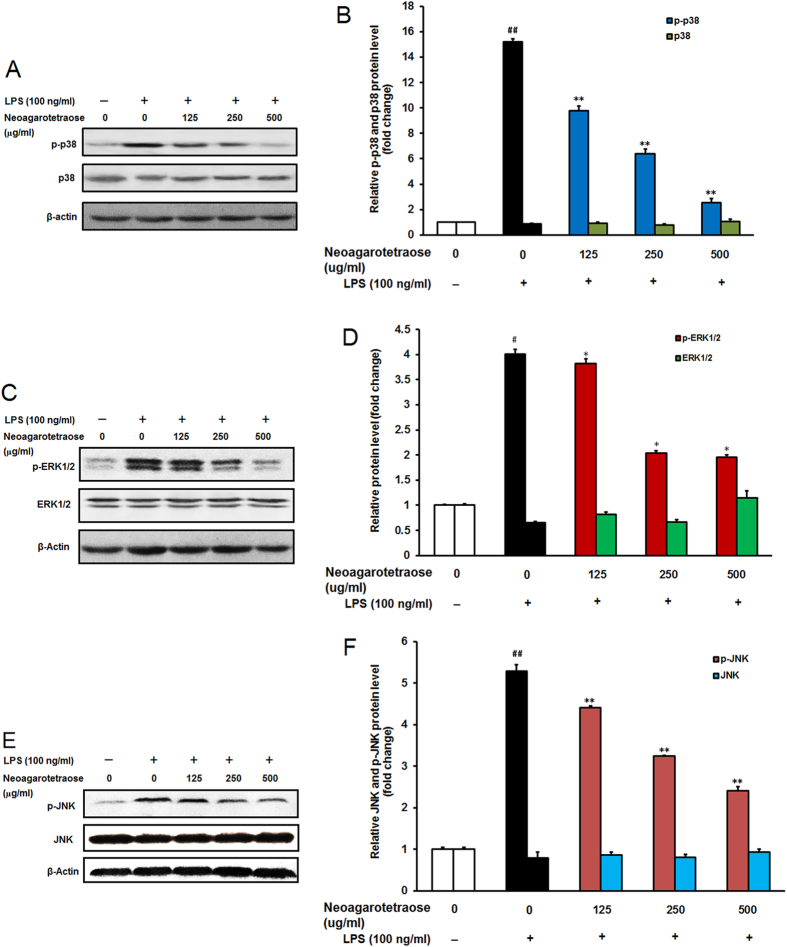
Effect of neoagarotetraose on the activation of MAPK pathway in RAW264.7 cells. (**A**) After pretreated with neoagarotetraose (125, 250, 500 μg/ml) for 2 h, RAW264.7 cells were exposed to 100 ng LPS for 1 h. Then the expression levels of p38MAPK and phosphorylated p38MAPK were detected by western blot, respectively. Blots were also probed for β-actin as loading controls. The result shown is a representative of three separate experiments with similar results. (**B**) Quantification of immunoblot for the ratio of p38MAPK or phosphorylated p38MAPK to β-actin. The ratio for non-treated control cells was assigned a value of 1.0 and the data presented as mean ± SD (n = 3). Significance: ^##^P < 0.01 vs. normal control; **P < 0.01 vs. LPS treated control. (**C**) After treatment, the expression levels of ERK1/2 and phosphorylated ERK1/2 were detected by western blot, respectively. (**D**) Quantification of immunoblot for the ratio of total ERK1/2 or phosphorylated ERK1/2 to β-actin. The ratio for non-treated normal control cells was assigned a value of 1.0 and the data presented as mean ± SD (n = 3). Significance: ^#^P < 0.05 vs. normal control; *P < 0.05 vs. LPS treated control.

It was reported that LPS treatment could stimulate cellular MAPK pathways including p38MAPK, Ras/MEK/ERK, and JNK pathways, which was associated with the inflammation responses^20^,^21^,^22^,^23^,^24^. Thus, we further investigated whether the inhibition effects of neoagarotetraose on inflammation responses were related to the MAPK signaling pathway. Firstly, the influence of neoagarotetraose on phosphorylation of p38MAPK was evaluated by western blot assay. As shown in Fig. 5A,B, LPS stimulation significantly increased the phosphorylation of p38MAPK in macrophage RAW264.7 cells compared to the normal control group (P < 0.01). Pretreatment of neoagarotetraose (125, 250, 500 μg/ml) significantly suppressed the phosphorylation of p38MAPK from 15.2 to about 9.8, 6.4 and 2.6-fold of the normal control, respectively (P < 0.01), as compared to that in the LPS treated control group (Fig. 5B). However, neoagarotetraose did not significantly reduce the total levels of p38MAPK in LPS-activated macrophages (Fig. 5A,B).

Moreover, the influence of neoagarotetraose on Ras/MEK/ERK pathway was also evaluated by western blot. The results showed that LPS treatment significantly increased the phosphorylation of ERK1/2 compared to the normal control group (P < 0.05) but the total level of ERK1/2 in RAW264.7 cells did not significantly change (Fig. 5C,D). Pretreatment of neoagarotetraose (125, 250, 500 μg/ml) significantly reduced the phosphorylation of ERK1/2 from 3.9 to about 3.6, 2.1 and 2.0-fold of the normal control, respectively (P < 0.05) (Fig. 5D). However, neoagarotetraose did not significantly decrease the total levels of ERK1/2, which suggested that neoagarotetraose may also inhibit the activation of Ras/MEK/ERK pathway. Taken together, MAPK signal pathways might be effectively blocked by neoagarotetraose in LPS stimulated RAW264.7 cells.

Furthermore, the influence of neoagarotetraose on JNK pathway was also evaluated by western blot. The results showed that LPS treatment significantly increased the phosphorylation of JNK compared to the normal control group (P < 0.01) but the total level of JNK in RAW cells did not significantly change (Fig. 5E,F). Pretreatment of neoagarotetraose (125, 250, 500 μg/ml) significantly reduced the phosphorylation of JNK from 5.3 to about 4.4, 3.2 and 2.4-fold of the normal control, respectively (P < 0.01) (Fig. 5F). However, neoagarotetraose did not significantly influence the total levels of JNK, which suggested that neoagarotetraose may also inhibit the activation of JNK pathway. Taken together, MAPK signal pathways might be effectively blocked by neoagarotetraose in LPS stimulated RAW264.7 cells.

### Effects of neoagarotetraose on NF-κB pathway in LPS-stimulated RAW264.7 cells

**Figure 6:**
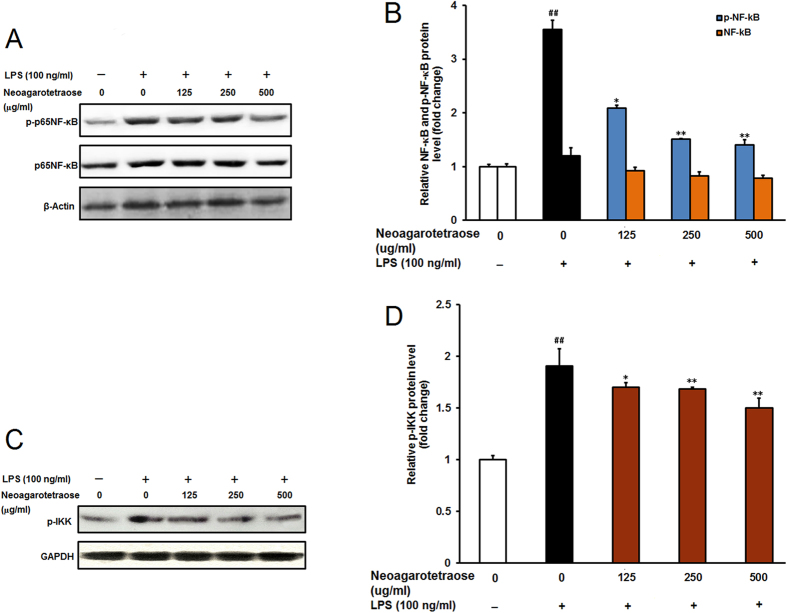
Effect of neoagarotetraose on the activation of NF-κB pathway in RAW264.7 cells. (**A**) After pretreated with neoagarotetraose (125, 250, 500 μg/ml) for 2 h, the LPS (100 ng) was added to cells and incubated for 1 h. Then the expression levels of phosphorylated NF-κB and total NF-κB were detected by western blot analysis, respectively. Blots were also probed for β-actin as loading controls. The result shown is a representative of three separate experiments with similar results. (**B**) Quantification of immunoblot for the ratio of total NF-κB and phosphorylated NF-κB to β-actin. The ratio for non-treated normal control cells was assigned a value of 1.0 and the data presented as mean ± SD (n = 3). Significance: ^##^P < 0.01 vs. normal control; *P < 0.05, **P < 0.01 vs. LPS treated control. (**C**) After pretreated with neoagarotetraose (125, 250, 500 μg/ml) for 2 h, the LPS (100 ng) was added to cells and incubated for 1 h. Then the levels of phosphorylated IKK were detected by western blot. Blots were also probed for GAPDH as loading controls. (**D**) Quantification of immunoblot for the ratio of phosphorylated IKK to GAPDH. The ratio for non-treated normal control cells was assigned a value of 1.0 and the data presented as mean ± SD (n = 3). Significance: ^##^P < 0.01 vs. normal control; *P < 0.05, **P < 0.01 vs. LPS treated control.

NF-κB pathway was reported to be related to the immune responses and inflammation responses in macrophages^25^,^26^. The activation of NF-κB is often required for the upregulation of pro-inflammatory mediators, such as, iNOS, TNF-α, and IL-6, in LPS-induced RAW264.7 macrophages, so the effect of neoagarotetraose on LPS-induced NF-κB activation was also evaluated by western blot. As shown in Fig. 6A,B, LPS stimulation significantly increased the phosphorylation of NF-κB p65 subunit in macrophage RAW264.7 cells compared to the normal control group (P < 0.01). Pretreatment of neoagarotetraose (125, 250, 500 μg/ml) significantly suppressed the phosphorylation of p65 from 2.5 to about 2.1, 1.8 and 1.4-fold of normal control group, respectively (P < 0.05) (Fig. 6B). However, neoagarotetraose could not significantly reduce the total levels of p65 in LPS-activated macrophages (Fig. 6A,B). Moreover, the influence of neoagarotetraose on phosphorylated IKK which was the upstream signal molecule of NF-κB was also evaluated (Fig. 6C,D). The results showed that LPS treatment significantly increased the phosphorylation of IKK compared to the normal control group (P < 0.01) (Fig. 6C). Pretreatment of neoagarotetraose (125, 250, 500 μg/ml) significantly reduced the phosphorylation of IKK from 1.9 to about 1.7, 1.6 and 1.5-fold of the normal control, respectively (P < 0.05) (Fig. 6D). Therefore, these results indicated a crucial role of NF-κB signaling in the anti-inflammation actions of neoagarotetraose.

In summary, neoagarotetraose may inhibit LPS induced inflammation responses through downregulating the MAPK and NF-κB pathways.

## Discussion

Recently, agaro-oligosaccharides have been reported to possess a variety of physiological activities, such as antioxidative activities and anti-inflammation effects^12^,^14^, suggesting that these oligosaccharides have great potential in development of functional foods. In the present study, the inhibitory effects and mechanisms of neoagaro-oligosaccharides against LPS-induced inflammatory response were investigated. The results showed that neoagarotetraose significantly inhibited LPS-induced inflammatory responses in RAW264.7 cells. The inhibition action may be due to the reduction of LPS-induced iNOS and IL-1β expression through downregulating both MAPK and NF-κB signaling pathways.

Inflammation is a host response to infectious microbes or injured tissues^1^,^27^ and involves recruitment and activation of neutrophils and macrophages^2^. During this process, some toxins such as LPS can stimulate the macrophages to induce a high production of NO by the inducible enzyme iNOS^28^,^29^. Herein, we found that neoagarotetraose significantly inhibited the mRNA expression of iNOS and IL-1β in LPS induced RAW264.7 cells, and, thus, inhibited the production and release of NO (Fig. 4). In addition, neoagarotetraose also concentration-dependently inhibited the expression of TNF-α and IL-6 at transcription level, and reduced the production and secretion of TNF-α and IL-6 in LPS stimulated cells (Fig. 3). Therefore, neoagarotetraose possessed inhibition actions on the production of key mediators in inflammation such as iNOS, TNF-α and other cytokines in LPS-stimulated RAW 264.7 macrophages.

It was reported that LPS treatment can stimulate cellular MAPK pathways including p38MAPK, Ras/MEK/ERK, and JNK pathways, which are associated with the inflammation responses^20^,^21^,^22^,^23^,^24^. In this study, neoagarotetraose oligosaccharide monomers inhibited the activation of p38MAPK, ERK1/2 and JNK in a dose-dependent manner in LPS-stimulated RAW264.7 cells, suggesting that neoagarotetraose may inhibit inflammation responses mainly through downregulating Ras/MEK/ERK, p38MAPK and JNK signaling pathways. It was reported that JNK signaling regulates the expression of iNOS, whereas Ras/MEK/ERK and p38MAPK signaling upregulate the production of iNOS and proinflammatory cytokines such as TNF-α and IL-6 in LPS-stimulated macrophages^20^,^21^. Thus, neoagarotetraose may inhibit MAPK signaling pathways to reduce the production of proinflammatory cytokines.

NF-κB pathway was reported to be related to the immune responses and inflammation responses in macrophages^25^,^26^. The activation of NF-κB which mediated by the NF-κB translocation dependent pathway^30^ or the phosphorylation of MAPK signaling^31^ is required for the upregulation of pro-inflammatory mediators in LPS-induced RAW264.7 cells. In the present study, we found that neoagarotetraose significantly decreased the LPS-induced phosphorylation of p65NF-κB rather than the expression level of total NF-κB. Moreover, neoagarotetraose also significantly inhibited the phosphorylation of IKK in LPS treated RAW cells (Fig. 6), suggesting that neoagarotetraose may also inhibit NF-κB pathway. Thus, neoagarotetraose may be able to inhibit both MAPK and NF-κB signaling pathways in LPS-induced RAW264.7 cells. Moreover, some anti-inflammatory peptides were reported to be able to suppress LPS-induced activation of macrophages through the interaction with LPS^32^. It was reported that low molecular-weight oligosaccharides could be internalized into cells to affect intracellular signal pathways^33^,^34^. Therefore, neoagarotetraose may inhibit LPS-induced inflammatory responses through direct interaction with LPS or affecting the MAPK and NF-κB pathways in macrophages.

In conclusion, neoagaro-oligosaccharide monomers especially neoagarotetraose substantially suppressed the pro-inflammatory mediators such as iNOS and IL-1β as well as various cytokines (IL-6 and TNF-α) in LPS stimulated RAW264.7 cells by blocking both MAPK and NF-κB signaling pathways. Therefore, the neoagarotetraose merits further investigation as a novel therapeutic agent against inflammation related diseases in the future.

## Methods

### Preparation of neoagaro-oligosaccharide monomers

Neoagarobiose, neoagarotetraose, neoagarohexaose, neoagarooctaose, and neoagarodecaose (≥98% purity) (Fig. 1A) were prepared in our laboratory as described previously^15^,^16^,^17^,^18^. β-agarases used for preparation of neoagaro-oligosaccharides were all shown in Table 1. In brief, 100 ml 0.25% low-melting point agarose and corresponding 1600 U recombinant agarase were mixed and incubated at the optimum temperature for 48 h. Then the enzyme hydrolysis solution was heated in boiling water for 10 min and concentrated using vacuum-rotary evaporation at 55 °C. The concentrate was transferred into centrifuge tubes and mixed with 3 times volume of absolute ethanol. The supernatant was collected by centrifugation at 10,000 rpm for 15 min at 4 °C and repeatedly concentrated to the neoagaro-oligosaccharide powder without moisture. The powder was dissolved in 1–3 ml ultrapure water and then extracted and purified by gel filtration using NH_4_HCO_3_ (0.5 mol/L) as eluent. The neoagaro-oligosaccharide monomers were detected by TLC, and the same component of each monomer was lyophilized and obtained eventually. The structures of compounds were characterized by High Resolution Mass Spectrometer (HRMS) analysis (Figure S1) (Supplementary Information).

### Reagents

LPS and MTT were purchased from Sigma–Aldrich (St. Louis, MO, USA). ELISA kit to NO was obtained from Beyotime (Nantong, Jiangsu, China). Antibodies to phospho-ERK1/2, p38, JNK, NF-κB, IKK, and antibodies to total p38, ERK1/2, JNK, NF-κB and β-actin, GAPDH were obtained from Cell Signaling Technology (Danvers, MA, USA). Alkaline phosphatase (AP) labeled secondary antibodies were purchased from Santa Cruz Biotechnology (Santa Cruz, CA, USA). ELISA kits to mouse iNOS, IL-1β, TNF-α and IL-6 were obtained from Dakewei (Shenzhen, China). All other reagents were of analytical grade.

### Cell culture

RAW264.7 cells were obtained from Cell Bank of Chinese Academy of Sciences (Shanghai, China) and grown in DMEM supplemented with 10% FBS and penicillin (100 U/ml)/streptomycin (100 μg/ml) at 37 °C in a 5% CO_2_ humidified incubator. Cells grown to 80% confluence were pretreated with neoagaro-oligosaccharide monomers (62.5, 125, 250 and 500 μg/ml) for 2 h and then treated with LPS (100 ng) for 16 h. Acetylsalicylic acid (25, 50, 100 and 200 μg/ml) was used as a positive control drug.

### Cytotoxicity assay

The cytotoxicity of neoagaro-oligosaccharides on RAW264.7 cells was measured by MTT assay^35^. The cells were cultured in 96-well plates at density of 1 × 10^4^ cells/well. After 24 h, the cells were added with 62.5, 125, 250, 500 and 1000 μg/ml of neoagaro-oligosaccharide monomers and then incubated at 37 °C for 24 h. Then the MTT solution was added to each well and further incubated for 4 h at 37 °C. The medium was discarded and DMSO was added to dissolve the formazan dye. The optical density was determined at 540 nm. The cell viability was expressed as a percentage of non-treated control.

### Measurement of nitrite in culture media

The nitrite accumulated in culture medium was measured as an indication of nitric oxide (NO) production based on the Griess reaction as previously described^8^. Briefly, on a 96-well plate, the cell supernatant (100 μl) was mixed with the Griess reagent (100 μl), which was prepared as follows: 1:1 (v/v) of 0.1% N-1-naphthyl-ethylenediamine in distilled water and 1% sulfanilamide in 5% phosphoric acid. After 10 min incubation, the absorbance was measured at 550 nm, and the amount of nitrite was calculated from the NaNO_2_ standard curve.

### Reverse transcription-polymerase chain reaction (RT-PCR)

Total RNA in RAW264.7 cells was extracted using trizol reagent (Invitrogen, USA). One microgram of RNA was firstly reverse-transcribed into cDNA using M-MLV first strand cDNA synthesis kit (Omega, USA). Then the products were subjected to quantitative PCR assay using the following primers^36^: TNF-α mRNA, 5′-CTCTTCTCATTCCTGCTTG-3′ and 5′-CTCCACTTGGTGGTTTGT-3′; IL-6 mRNA, 5′-CACAGAAGGAGTGGCTAA-3′ and 5′-CCATAACGCACTAGGTTT-3′; iNOS mRNA, 5′-CACGGACGAGACGGATAG-3′ and 5′-TGCGACAGCAGGAAGG-3′; IL-1β mRNA, 5′-GGTACATCAGCACCTCAC-3′ and 5′-AAACAGTCCAGCCCATAC-3′; β-actin mRNA, 5′-CACTGTGCCCATCTACGA-3′ and 5′-TGATGTCACGCACGATTT-3′. The relative amounts of these mRNAs were determined using the comparative (2^−ΔΔCT^) method, as previously described^37^.

### Western blot assay

Western blot analysis was performed as described previously^8^. In brief, cell lysates were separated on SDS-polyacrylamide gels, and transferred onto nitrocellulose membranes. Then the membranes were probed with the antibodies to phospho-NF-κB, NF-κB, phospho-ERK1/2, ERK1/2, phospho-p38, p38, phospho-JNK, JNK, phospho-IKK, or β-actin and GAPDH proteins overnight at 4 °C. After that, the membranes were incubated with Alkaline Phosphatase-conjugated secondary antibodies and visualized by incubating with the developing solution [p-nitro blue tetrazolium chloride (NBT) and 5-bromo-4-chloro-3-indolyl phosphate toluidine (BCIP)] at room temperature for 30 min. The relative densities of protein bands were determined by using Image J (NIH) v.1.33 u (USA).

### Statistical analysis

All data are representative of at least three independent experiments. All data are represented as the mean ± S.D. Statistical significance was calculated by SPSS 10.0 software using the two-tailed unpaired t-test analysis and the variance analysis (ANOVA). P < 0.05 was considered statistically significant.

## Additional Information

**How to cite this article**: Wang, W. *et al*. Neoagaro-oligosaccharide monomers inhibit inflammation in LPS-stimulated macrophages through suppression of MAPK and NF-κB pathways. *Sci. Rep.* **7**, 44252; doi: 10.1038/srep44252 (2017).

**Publisher's note:** Springer Nature remains neutral with regard to jurisdictional claims in published maps and institutional affiliations.

## Supplementary Material

Supplementary Information

## Figures and Tables

**Table 1:** Enzymes used for preparation of neoagaro-oligosaccharides in this work.

Enzymes	Source	Product	Reference
AgWH50C	*Agarivorans gilvus* WH0801	Neoagarobiose	16
AgWH50A	*Agarivorans gilvus* WH0801	Neoagarotetraose	17
AgWH16	*Agarivorans gilvus* WH0801	Neoagarohexaose	Unpublished
Agarase-a	*Agarivorans albus* OAY02	Neoagarooctaose	15,18

## References

[b1] Oseguera-Toledo M. E., de Mejia E. G., Dia V. P. & Amaya-Llano S. L. Common bean (Phaseolus vulgaris L.) hydrolysates inhibit inflammation in LPS-induced macrophages through suppression of NF-κB pathways. Food Chemistry 127, 1175–1185 (2011). 25214111 10.1016/j.foodchem.2011.01.121

[b2] Li D. Y., Xue M. Y., Geng Z. R. & Chen P. Y. The suppressive effects of Bursopentine (BP5) on oxidative stress and NF-κB activation in lipopolysaccharide-activated murine peritoneal macrophages. Cellular Physiology and Biochemistry 29(1–2), 9–20 (2012). 22415070 10.1159/000337581

[b3] Kim K. N. . Fucoxanthin inhibits the inflammatory response by suppressing the activation of NF-kappaB and MAPKs in lipopolysaccharide-induced RAW264.7 macrophages. European Journal of Pharmacology 649(1–3), 369–375 (2010). 20868674 10.1016/j.ejphar.2010.09.032

[b4] Martinez F. O. Regulators of macrophage activation. European Journal of Immunology 41(6), 1531–1534 (2011). 21607943 10.1002/eji.201141670

[b5] Akira S. & Takeda K. Toll-like receptor signalling. Nature Reviews Immunology 4(7), 499–511 (2004). 10.1038/nri1391 15229469

[b6] Heller R. A. . Discovery and analysis of inflammatory disease-related genes using cDNA microarrays. Proceeding of the National Academy of Sciences of the United States of America 94(6), 2150–2155 (1997). 10.1073/pnas.94.6.2150 PMC20056 9122163

[b7] Janeway C. A. Jr. & Medzhitov R. Innate immune recognition. Annual Review of Immunology 20, 197–216 (2002). 10.1146/annurev.immunol.20.083001.084359 11861602

[b8] Yoo M. S. . Fucosterol isolated from Undaria pinnatifida inhibits lipopolysaccharide-induced production of nitric oxide and pro-inflammatory cytokines via the inactivation of nuclear factor-κB and p38 mitogen-activated protein kinase in RAW264.7 macrophages. Food Chemistry 135, 967–975 (2012). 22953812 10.1016/j.foodchem.2012.05.039

[b9] Araki C. Seaweed polysaccharides. In carbohydrate chemistry of substances of biological interest; Wolfrom, M. L. Ed.; Pergamon Press: London, United Kingdom **1959**, pp 15–30.

[b10] Temuujin U., Chi W. J., Lee S. Y., Chang Y. K. & Hong S. K. Overexpression and biochemical characterization of DagA from Streptomyces coelicolor A3(2): an endo-type β-agarase producing neoagarotetraose and neoagarohexaose. Applied Microbiology and Biotechnology 92, 749–759 (2011). 21655986 10.1007/s00253-011-3347-7

[b11] Fu X. T. & Kim S. M. Agarase: Review of major sources, categories, purification method, enzyme characteristics and applications. Marine Drugs 8, 200–218 (2010). 20161978 10.3390/md8010200 PMC2817930

[b12] Wang J. X., Mou H. J., Jiang X. L. & Guan H. S. Biological activities of a neutral water-soluble agar polysaccharide prepared by agarose degradation. High Technology Letters. 11, 415–420 (2005).

[b13] Ohta Y. . Enzymatic properties and nucleotide and amino acid sequences of a thermostable β-agarase from a novel species of deep-sea *Microbulbifer*. Applied Microbiology and Biotechnology 64, 505–514 (2004). 15088129 10.1007/s00253-004-1573-y

[b14] Yun E. J. . Enzymatic production of 3,6-anhydro-L-galactose from agarose and its purification and *in vitro* skin whitening and anti-inflammatory activities. Applied Microbiology and Biotechnology 97, 2961–2970 (2013). 22678025 10.1007/s00253-012-4184-z

[b15] Li J. . A simple method of preparing diverse neoagaro-oligosaccharides with β-agarase. Carbohydrate Research. 342, 1030–1033 (2007). 17359946 10.1016/j.carres.2007.02.008

[b16] Liu N., Mao X. Z., Du Z. J., Mu B. Z. & Wei D. Z. Cloning and characterisation of a novel neoagarotetraose-forming-β-agarase, AgWH50A from Agarivorans gilvus WH0801. Carbohydrate Research 388, 147–151 (2014). 24642364 10.1016/j.carres.2014.02.019

[b17] Liu N., Mao X. Z., Yang M., Mu B. Z. & Wei D. Z. Gene cloning, expression and characterisation of a new b-agarase, AgWH50C, producing neoagarobiose from Agarivorans gilvus WH0801. World Journal of Microbiology and Biotechnology 30, 1691–1698 (2014). 24395600 10.1007/s11274-013-1591-y

[b18] Yang M., Mao X. Z., Liu N., Qiu Y. Q. & Xue C. H. Purification and characterization of two agarases from Agarivorans albus OAY02. Process Biochemistry 49, 905–912 (2014).

[b19] Guha M. & Mackman N. LPS induction of gene expression in human monocytes. Cell Signal 13, 85–94 (2001). 11257452 10.1016/s0898-6568(00)00149-2

[b20] Ajizian S. J., English B. K. & Meals E. A. Specific inhibitors of p38 and extracellular signal regulated kinase mitogen-activated protein kinase pathways block inducible nitric oxide synthase and tumor necrosis factor accumulation in murine macrophages stimulated with lipopolysaccharide and interferon-γ. Journal of Infectious Diseases 179, 939–944 (1999). 10068590 10.1086/314659

[b21] Choi H. J. . Ikarisoside A inhibits inducible nitric oxide synthase in lipopolysaccharide-stimulated RAW 264.7 cells via p38 kinase and nuclear factor-kappaB signaling pathways. European Journal of Pharmacology 601(1–3), 171–178 (2008). 18929556 10.1016/j.ejphar.2008.09.032

[b22] Schindler J. F., Monahan J. B. & Smith W. G. P38 pathway kinases as anti-inflammatory drug targets. Journal of Dental Reserch 86(9), 800–811 (2007). 10.1177/154405910708600902 17720847

[b23] Xiao Z. Y. . Inhibitory effect of linomide on lipopolysaccharide-induced proinflammatory cytokine tumor necrosis factor-α production in RAW264.7 macrophages through suppression of NF-κB, p38, and JNK activation. Immunology Letters 114, 81–85 (2007). 17964662 10.1016/j.imlet.2007.09.001

[b24] Chan E. D. & Riches D. W. IFN-γ+ LPS induction of iNOS is modulated by ERK, JNK/SAPK, and p38 (mapk) in a mouse macrophage cell line. American journal of physiology-cell physiology 280, C441–C450 (2001). 11171562 10.1152/ajpcell.2001.280.3.C441

[b25] García-Lafuente A. . *In vitro* anti-inflammatory activity of phenolic rich extracts from white and red common beans. Food Chemistry 161, 216–223 (2014). 24837943 10.1016/j.foodchem.2014.04.004

[b26] Liu F., Morris S., Epps J. & Carroll R. Demonstration of an activation regulated NF-kappaB/I-kappaB alpha complex in human platelets. Thrombosis Research 106(4–5), 199–203 (2002). 12297126 10.1016/s0049-3848(02)00130-5

[b27] Coker R. K. & Laurent G. J. Pulmonary fibrosis: cytokines in the balance. The European Respiratory Journal. 11, 1218–1221 (1998). 9657557 10.1183/09031936.98.11061218

[b28] Boscá L., Zeini M., Través P. G. & Hortelano S. Nitric oxide and cell viability in inflammatory cells: a role for NO in macrophage function and fate. Toxicology 208, 249–258 (2005). 15691589 10.1016/j.tox.2004.11.035

[b29] Gupta S. C., Sundaram C., Reuter S. & Aggarwal B. B. Inhibiting NF-κB activation by small molecules as a therapeutic strategy. Biochimica et Biophysica Acta 1799, 775–787 (2010). 20493977 10.1016/j.bbagrm.2010.05.004 PMC2955987

[b30] Pan M. H., Lin-Shiau S. Y. & Lin J. K. Comparative studies on the suppression of nitric oxide synthase by curcumin and its hydrogenated metabolites through down-regulation of Ikappa B kinase and NF-kappa B activation in macrophages. Biochemical Pharmacology 60(11), 1665–1676 (2000). 11077049 10.1016/s0006-2952(00)00489-5

[b31] Craig R. . P38 MAPK and NF-kappa B collaborate to induce interleukin-6 gene expression and release. Evidence for a cytoprotectiveautocrine signaling pathway in a cardiac myocyte model system. Journal of Biological Chemistry 275(31), 23814–23824 (2000). 10781614 10.1074/jbc.M909695199

[b32] Nagaoka I. . Cathelicidin family of antibacterial peptides CAP18 and CAP11 inhibit the expression of TNF-alpha by blocking the binding of LPS to CD14(+) cells. Journal of Immunology 167(6), 3329–3338 (2001). 10.4049/jimmunol.167.6.3329 11544322

[b33] Wang W. . *In vitro* inhibitory effect of carrageenan oligosaccharide on influenza A H1N1 virus. Antiviral Research 92, 237–246 (2011). 21867732 10.1016/j.antiviral.2011.08.010

[b34] Hao C. . Insulin sensitizing effects of oligomannuronate-chromium (III) complexes in C2C12 skeletal muscle cells. PLoS One 6, e24598 (2011). 21935427 10.1371/journal.pone.0024598 PMC3174176

[b35] Ferrari M., Fornasiero M. C. & Isetta A. M. MTT colorimetric assay for testing macrophage cytotoxic activity *in vitro*. Journal of Immunological Methods 131, 165–172 (1990). 2391427 10.1016/0022-1759(90)90187-z

[b36] Fan X. L. . Trilobatin attenuates the LPS-mediated inflammatory response by suppressing the NF-κB signaling pathway. Food Chemistry 166, 609–615 (2015). 25053100 10.1016/j.foodchem.2014.06.022

[b37] Livak K. J. & Schmittgen T. D. Analysis of relative gene expression data using Real-Time quantitative PCR and the 2^−ΔΔCT^ method. Method 25(4), 402–408 (2001). 10.1006/meth.2001.1262 11846609

