# Supplementary material for: Neoagaro-oligosaccharide monomers inhibit inflammation in LPS-stimulated macrophages through suppression of MAPK and NF-κB pathways
Source: Sci Rep. 2017 Mar 7;7:44252. doi: 10.1038/srep44252 (PMC5339798; doi:10.1038/srep44252)
Supplement: Supplementary Information [file srep44252-s1.pdf]

Supplementary Information

The title of the manuscript

Neoagaro-oligosaccharide monomers inhibit inflammation in LPS-stimulated macrophages through suppression of MAPK and NF-κB pathways

Wei Wang[b, #], Pei Liu[a, #], Cui Hao[c], Lijuan Wu[b], Wenjin Wan[d], Xiangzhao Mao[a,*]

[a] College of Food Science and Engineering, Ocean University of China, Qingdao, 266003, China.

[b] Key Laboratory of Marine Drugs of Ministry of Education, School of Medicine and Pharmacy, Ocean University of China, Qingdao, 266003, China.

[c] Institute of Cerebrovascular Diseases, Affiliated Hospital of Qingdao University Medical College, Qingdao, 266003, China.

[d] Department of Biology, Hong Kong Baptist University, Hong Kong, China.

***Corresponding author:**

Tel.: +86 532 8203 1360; fax: +86 532 2272 1360; E-mail: xzhmao@ouc.edu.cn (X.Z.M.)

# These authors contributed equally to this paper.

Neoagarobiose (NA2)

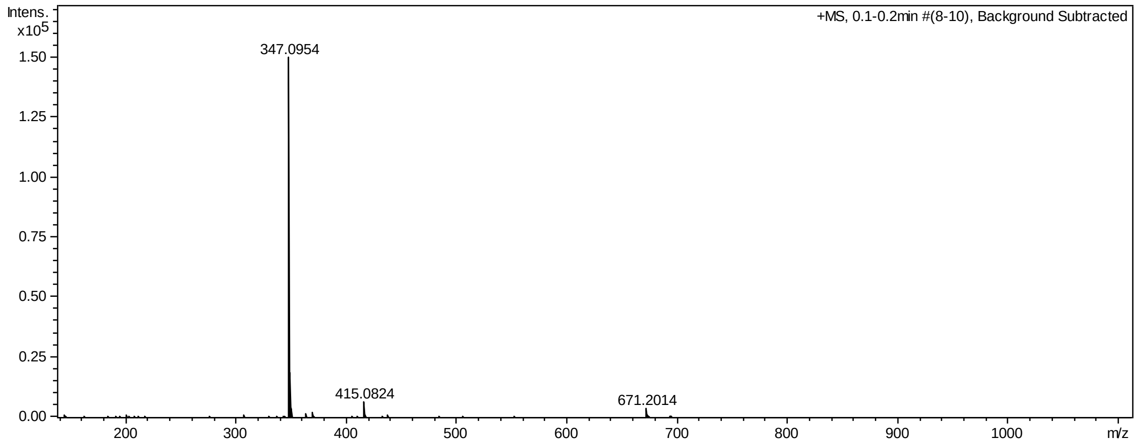

Neoagarotetraose (NA4)

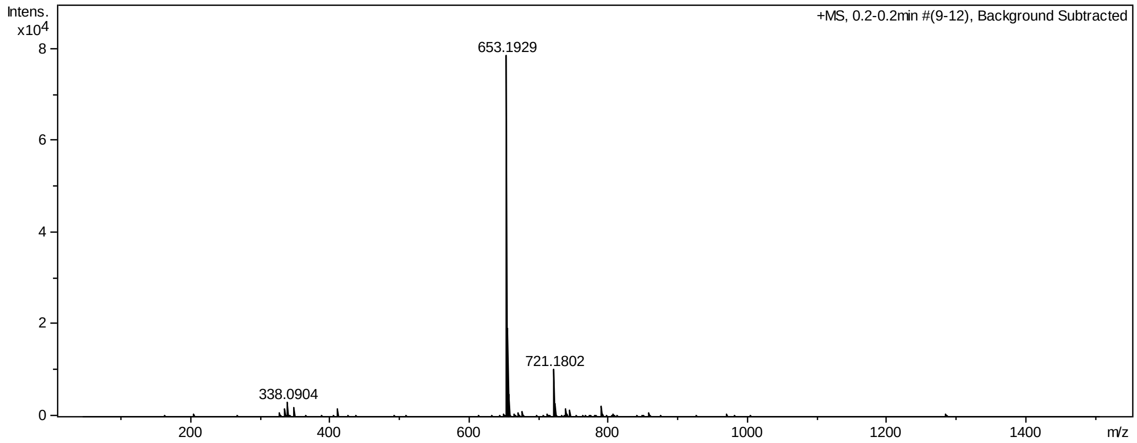

Neoagarohexaose (NA6)

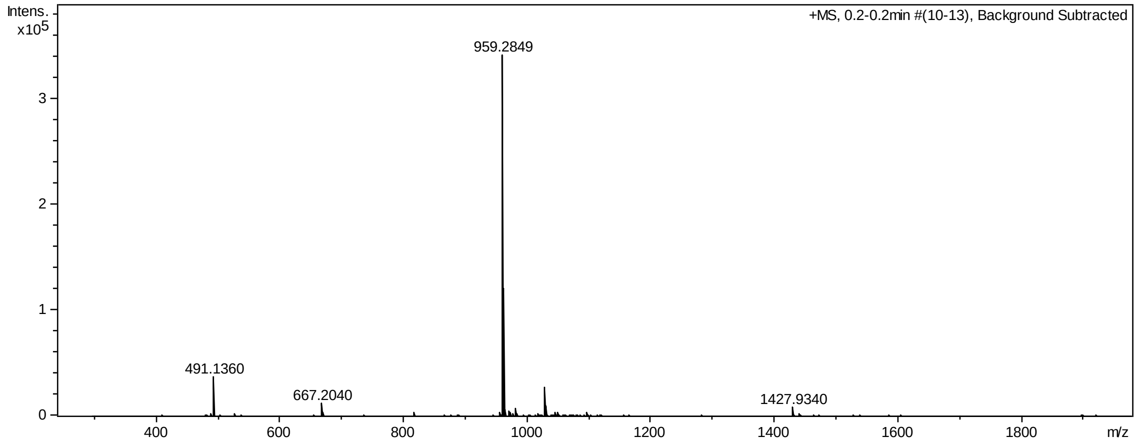

Neoagarooctaose (NA8)

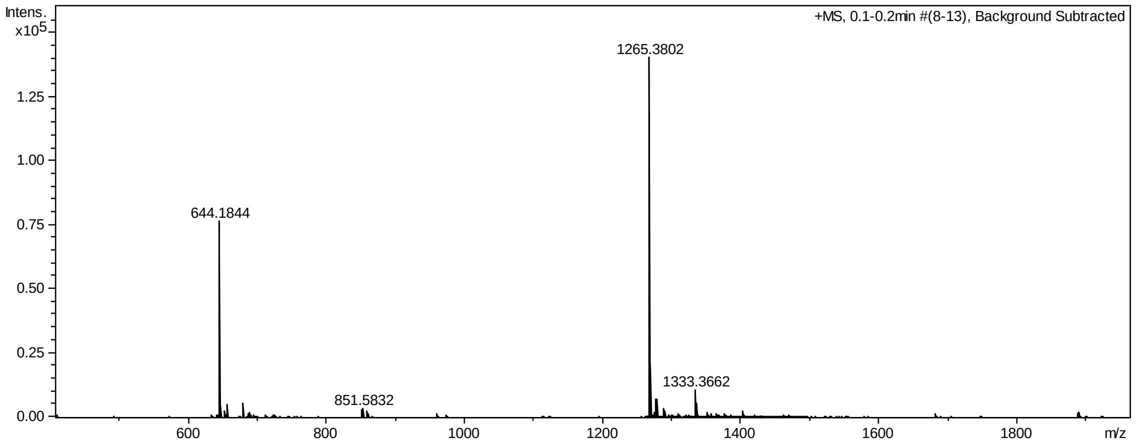

Neoagarodecaose (NA10)

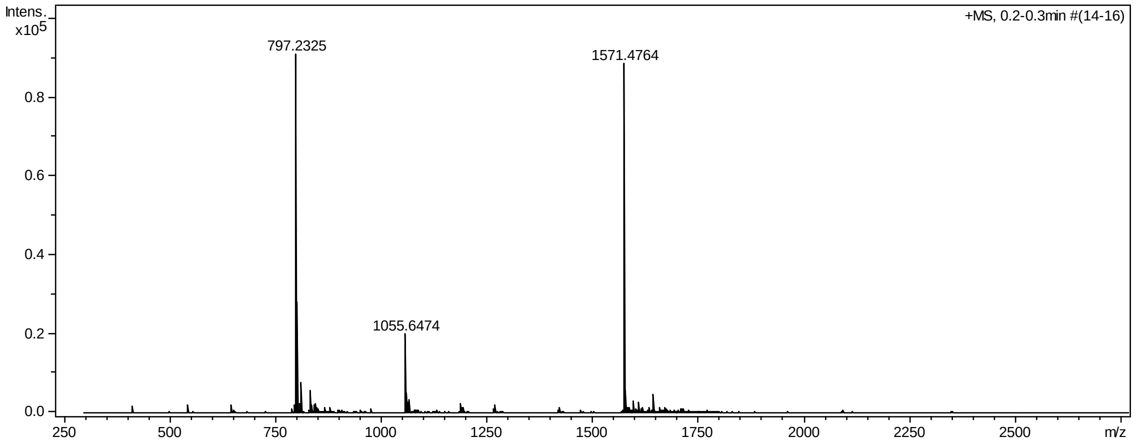

Fig. S1. MALDI-TOF mass spectrum of neoagaro-oligosaccharide monomers. Neoagarobiose (**NA2**), 324 Da + 23 Da (Na+) was marked; neoagarotetraose (**NA4**), 630 Da + 23 Da (Na+) was marked; neoagarohexaose (**NA6**), 936 Da + 23 Da (Na+) was marked; neoagarooctaose (**NA8**), 1242 Da + 23 Da (Na+) was marked; neoagarodecaose (**NA10**), 1548 Da + 23 Da (Na+) was marked.
